# Does the Adult Human Ciliary Body Epithelium Contain “True” Retinal Stem Cells?

**DOI:** 10.1155/2013/531579

**Published:** 2013-10-28

**Authors:** Rebecca Frøen, Erik O. Johnsen, Bjørn Nicolaissen, Andrea Facskó, Goran Petrovski, Morten C. Moe

**Affiliations:** ^1^Department of Ophthalmology, Oslo University Hospital, Pb 4950 Nydalen, 0407 Oslo, Norway; ^2^Department of Ophthalmology, Faculty of Medicine, University of Szeged, Korányi fasor 10-11, Szeged 6720, Hungary; ^3^Stem Cells and Eye Research Laboratory, Department of Biochemistry and Molecular Biology, Medical and Health Science Center and Apoptosis and Genomics Research Group of the Hungarian Academy of Sciences, University of Debrecen, Nagyerdei krt. 98, Debrecen 4032, Hungary

## Abstract

Recent reports of retinal stem cells being present in several locations of the adult eye have sparked great hopes that they may be used to treat the millions of people worldwide who suffer from blindness as a result of retinal disease or injury. A population of proliferative cells derived from the ciliary body epithelium (CE) has been considered one of the prime stem cell candidates, and as such they have received much attention in recent years. However, the true nature of these cells in the adult human eye has still not been fully elucidated, and the stem cell claim has become increasingly controversial in light of new and conflicting reports. In this paper, we will try to answer the question of whether the available evidence is strong enough for the research community to conclude that the adult human CE indeed harbors stem cells.

## 1. Introduction

In the retina, light is translated into electrical impulses that are processed and transmitted further into the brain through a complex neuronal chain. This sensory pathway is damaged in common eye diseases such as retinal degenerative diseases, diabetic retinopathy, arterial occlusions, traumas, and glaucoma. Stem cell-based therapies still hold great promise to treat several neurodegenerative diseases and/or injuries, and the retina may be an ideal candidate for regenerative medicine due to its relatively small size and immunity, as well as recent discoveries in retinal microsurgery and visualization [1]. There are three main categories of human stem cells which are currently being investigated for retinal regenerative therapy: embryonic stem cells (ESCs) [2], induced pluripotent stem cells (iPS cells) [3], and somatic or adult neural stem cells (NSCs) [1, 4]. One of the putative advantages of adult NSCs is the possibility for autologous transplantation without reprogramming, whereby NSCs may be harvested from adult patients, expanded or modified *in vitro*, and re-transplanted into the original patient [5]. However, most studies regarding isolation and characterization of NSCs in the adult eye have until recently been performed in lower vertebrates and rodents [4]. In this review, we will focus on the adult human eye.

The neuroretina—like the rest of the central nervous system (CNS)—is considered to have limited regenerative potential in adult humans, and severe injuries can lead to permanent damage for which currently there are no definitive curative treatment options. Until the 1990s, it was a central dogma of neuroscience that no new neurons could be formed in the adult human CNS. This doctrine was best formulated in the words of the histologist Ramon y Cajal: “Once the development was ended, the fountains of growth and regeneration of the axons and dendrites dried up irrevocably. In the adult centers, the nerve paths are something fixed, ended, and immutable. Everything may die, nothing may be regenerated. It is for the science of the future to change, if possible, this harsh decree.” [6]. However, despite this dogma, researchers have continuously tried to identify NSCs in humans that are both able to self-renew and differentiate into functional retinal cell types to treat patients with retinal disorders, and one of the key scientific questions has been whether such NSCs exist in the patient's own eye. In this search for retinal stem cells (RSCs), the ciliary body epithelium (CE) has been considered as one of the prime niches. There is also evidence that both Müller glia [7–10] and retinal pigment epithelial (RPE) cells [11] can have properties of NSCs in the adult human eye, but these important topics will not be addressed in the current review.

## 2. The RSC Hypothesis

The development of the retina forms the theoretical background for the RSC hypothesis. During embryogenesis, the optic cup forms as a double-layered extension of the forebrain, with which it is continuous [12]. The inner layer of the optic cup differentiates into the neural retina centrally and into the nonpigmented layer of the CE, and iris peripherally, while the outer layer gives rise to three types of pigmented epithelial cells: RPE, pigmented CE and iris pigmented epithelium (IPE). Thus, all of these tissues—although diverse—share a common origin from multipotent NSCs and form a structural and developmental continuum with the brain. 

The second element that lead to the RSC hypothesis is the observation that in many lower vertebrates new retinal cells are constantly produced throughout life from multipotent cells residing in the ciliary marginal zone (CMZ) [13]. In mammals, this zone anatomically corresponds to the area around the peripheral margin of the retina and contains NSCs during retinogenesis. However, after birth, this stem cell population appears to have been depleted or remains in a quiescent state *in vivo *[8, 12]. Based on the above considerations, it was postulated that it is possible to isolate and propagate a rare/small population of quiescent RSCs from the adult mammalian CE. The first evidence to support this claim was put forward by Tropepe et al. and Ahmad et al. in 2000 [14, 15], and a number of studies on the proliferative and differentiation potential of these cells have since been performed to date [4, 8].

## 3. The Problem of Identifying a Stem Cell 

If a subpopulation of CE cells is to be labeled as stem cells, they must fulfill certain criteria. A stem cell is commonly defined as a cell that has the ability to (a) self-renew, (b) proliferate to form progenitor cells with a higher degree of lineage commitment, and (c) ultimately give rise to all the terminally differentiated and functional cells of the tissue from which it is derived [16]. In contrast to stem cells, progenitor cells could have a more restricted lineage potential. When trying to judge whether a population of cells meet these criteria, we face several problems. There are few, if any, genetic markers or morphological characteristics that precisely identify a stem cell as such. Thus, one can only conclude that stem cells are present in a tissue sample retrospectively, based on the functional criteria of proliferation, self-renewal, and production of differentiated cells [17]. 

**Figure 1:**
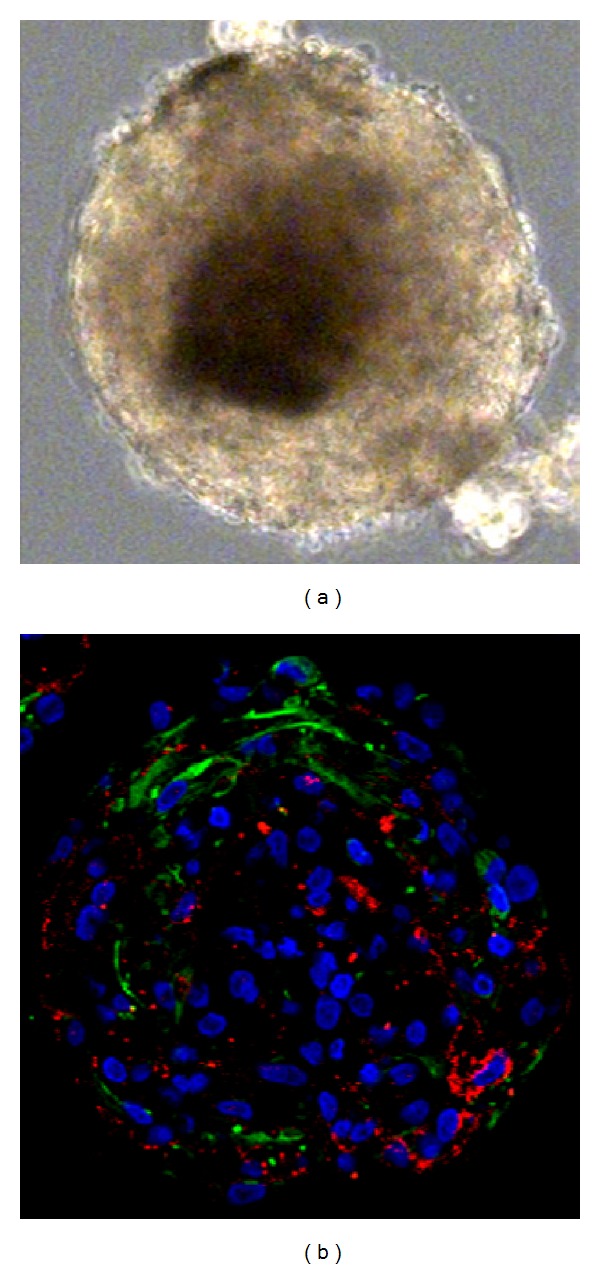
Light microscopy of secondary sphere derived from postmortem CE (a). Immunocytochemical analysis of secondary spheres derived from postmortem CE (b), costaining of Nestin (green) and Claudin-1 (red). Nuclear staining with Hoechst (blue).

When studying NSCs *in vitro*, it is common to use the neurosphere assay, first described by Reynolds and Weiss [18], where the tissue is prepared to form a single-cell suspension and cultured in a defined medium containing mitogens. After a few days, free-floating clusters of cells with a characteristicALLY rounded appearance, known as neurospheres, are formed (Figure 1(a)). These are thought to represent stem cells and their progeny [18]. Through repeated passaging, where the spheres are dissociated and replated as single cells, the stem/progenitor cell population may be expanded. This assay has also become an important method to strengthen the presence of a stem/progenitor cell population in a tissue. The continued formation of neurospheres over the course of many passages is interpreted as an expression of the stem cell's capacity for self-renewal and the expression of mature neural and glial markers as the stem cell's multipotency [17, 19].

However, the neurosphere assay has limitations. The population of cells within a sphere is heterogenous, consisting of cells at many different stages of differentiation and committed to different lineages [20–22]. Also, the neurosphere culturing method is sensitive to variations in factors such as cell density, concentrations of added mitogens, and number of passages. This can make it difficult to compare results between research groups and may account for the great variation in published results [16, 19]. *In vivo, *stem cells are thought to reside in a so-called stem cell niche, where their properties are carefully regulated by the structural and functional conditions of the extracellular matrix, cell-cell interactions, and complex signaling cascades [23]. The sphere may be viewed as an *in vitro *niche which provides different stimuli and cues to the cells therein. Thus, depending on the inherent plasticity of the cells, they may display different potency *in vitro *than they would be capable of *in vivo* [19]. It has recently been shown that sphere formation in culture, and CE spheres in particular, may grow nonclonally by incorporating other spheres and adherent cells. [24, 25]. Therefore, we can strictly only use sphere formation and repeated passaging as a test of the cells' ability to survive and proliferate in culture for extended periods of time, and not as a test of “stemcellness.” Lastly, evidence has also been presented that nonstem cells may be capable of forming clonogenic spheres in culture [26]. Since most of the evidence for the existence of RSCs in the adult ciliary body is based on the neurosphere assay, it is important to have a clear understanding of the benefits and limitations of this culture method. 

## 4. Evidence Favoring the Presence of RSCs in the Adult Human CE

Coles et al. attempted to culture cells isolated from the neural retina, pars plana and pars plicata of the ciliary body, RPE, and iris using the neurosphere assay and found that spheres were formed only from the ciliary body and iris. Of these, only spheres from the ciliary body could be passaged to form secondary spheres, indicating that only cells from this location exhibited the capacity for self-renewal. Multipotency was inferred by the immunohistochemical detection of markers for mature retinal cells of all lineages. Finally, cells were transplanted into developing mouse retinas, where a number of them showed signs of migration and integration into the host retina, as well as expression of mature retinal markers [27]. Mayer et al. found sphere-forming cells in both the pars plana and the neural retina itself (in contrast to the study cited above). These spheres consisted of cells expressing immature neuronal and glial markers. When exposed to differentiation conditions, a subset of cells expressing rhodopsin—a photoreceptor marker—was identified [28]. The same group later performed a study showing that adult human retina consistently gave rise to spheres in culture irrespective of age, sex, or postmortem time [29]. Xu et al. characterized spheres derived from the ciliary body, confirming earlier findings that they consist of proliferating cells that express certain immature neuronal and glial markers, while mature retinal markers could not be identified. Differentiation was not attempted [30]. 

Whilst the results of these studies partly support the adult RSC hypothesis, they have obvious weaknesses. The capability of sphere-forming CE cells for proliferation and self-renewal is well documented, but their multipotency is less so. To date, it has only been shown that these cells express certain mature retinal markers in culture. In order to conclude that functional retinal neurons have been formed, it would be necessary to demonstrate that they are postmitotic, have the correct morphology, and are capable of firing action potentials and releasing neurotransmitters [31]. Also, it is important to remember that these putative stem cells are derived from a nonneural tissue (but with neuroepithelial origin)—the CE. None of these papers investigated whether the CE-derived spheres contained a pure population of neural and glial cells—like neurospheres from the brain—or if they retained part of the epithelial phenotype of the tissue from which they were derived. This would have an important impact on their status as RSCs, as well on their potential use in cell-based therapy.

## 5. RSC or CE Cells?

Recently, several studies have questioned the existence of NSCs in the CE of the adult human eye [7, 9, 26, 34, 35]. Initially, we examined how the morphological characteristics and gene expression profiles of sphere-forming cells of the CE compared to those of brain-derived neural stem cells and found that CE spheres contained a population of proliferative epithelial-like cells with decreased expression of neural stem cell markers compared to CNS neurospheres [34] (Table 1). These results are partially in agreement with a recent study by Cicero et al., showing that although cells of the CE are able to clonally proliferate to form spheres and express certain markers of retinal stem/progenitor cells in culture, each cell still contained pigment and displayed membrane interdigitations and epithelial junctions, characteristic of differentiated ciliary epithelial phenotype [26]. Another recent study demonstrated that although CE cells in culture expressed significant levels of pluripotent and retinal progenitor markers, they consistently failed to differentiate into photoreceptors [35]. A study that separated the pigmented and nonpigmented CE found that only the nonpigmented CE proliferated to form spheres in culture, expressing high levels of epithelial markers, very limited numbers and levels of neural progenitor markers, and could not be induced to show signs of proper neural differentiation [7]. 

In light of this recent evidence, it is necessary to re-evaluate the RSC hypothesis regarding the adult human CE. The three stem cell functions described earlier: self-renewal, ability to form progenitor cells, and functional terminal differentiation are characteristically triggered by tissue injury. The self-renewal and proliferative capacity of CE cells is well documented [15, 27, 30, 34]. There is also little doubt that CE spheres contain a population of cells that display certain characteristics of neuroepithelial progenitors. Several studies have shown that CE spheres do express a range of immature neural and retinal markers [15, 27, 30, 34]. Importantly, we found that the spheres contain two distinct populations of cells: one Nestin^+^ and one Claudin-1^+^, while no double positive cells were detected (Figure 1(b)) [32]. This suggests that, in contrary to the conclusion drawn by Cicero et al., CE-derived spheres consist of a homogenous population of ciliary epithelial cells, they contain both epithelial cells and cells with a more neural progenitor-like phenotype.

**Figure 2:**
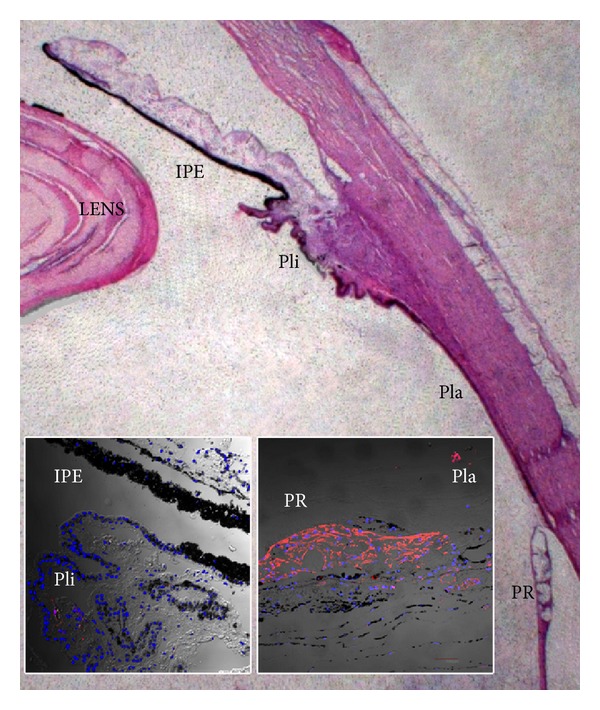
Light microscopic overview for illustrational purposes of the ciliary body epithelium (CE) consisting of pars plicata (Pli) and pars plana (Pla), as well as surrounding tissue including the lens and the iris pigmented epithelium (IPE) of the adult human eye. Almost complete absence of immunohistochemical Nestin-staining of IPE and Pli of the normal adult human eye (left inset), while there is intense Nestin-staining in the nonlaminated far peripheral retina (PR) and around peripheral cysts extending into the most posterior pars plana of the CE (right inset).

However, expression of certain progenitor markers *in vitro* is not sufficient evidence of the presence of true stem cells. Kohno et al. showed that CE-derived spheres initially consist of Nestin^−^ epithelial-like cells that begin to express Nestin during cultivation. These spheres had the ability to grow nonproliferatively by incorporating adherent Nestin^−^ cells, which then became Nestin^+^ [24]. It was later shown that CE cells rapidly upregulate this protein during the first 24 hrs in culture, before they have time to clonally proliferate [26]. Thus, it is possible that the cell population in CE spheres with neuroepithelial properties is not derived from true NSCs residing within the CE but rather from a trans-/de-differentiation process where CE cells respond to stem cell culture conditions by shifting their gene expression profile to an immature direction. In order to shed further light on this, we performed RT-PCR [32] (Table 1) and immunostaining [33] on wild-type adult human CE tissue and compared the expression of neural and epithelial genes to that of CE spheres. Immunostaining showed that most Nestin^+^ cells were found around peripheral cysts of the retina (Figure 2). While these cells also stained for the glial marker GFAP in the peripheral retina, they were GFAP^−^ in the adjacent proximal pars plana region [33]. Interestingly, we found no major clusters of Nestin^+^ cells or other putative NSC markers in the peripheral pars plana or pars plicata regions of the adult human CE (Figure 2).

The final test of a stem cell is in its capacity for producing differentiated cells. Some research groups have shown that CE cells can be induced to express markers of mature retinal neurons [14, 15, 27, 36–38], although only one of these studies was performed on human tissue. Moreover, others have shown recently that CE cells exposed to differentiation conditions tend to revert to a differentiated state of CE cells and not retinal cells [7, 26, 35]. This lack of consistence in results could be caused by differences in the culture protocols but could also be due to the fact that only the latter studies have looked for morphological and genetic characteristics of epithelial cells, while the earlier ones exclusively focused on neural and retinal markers. Ballios et al. recently demonstrated that a subpopulation of cells derived from the CE and sorted out on the basis of size and pigmentation criteria could be induced to express high levels of immature and mature photoreceptor markers in a sequential manner when cultured under specific differentiation conditions for extended periods of time (up to 40 days). These cells then no longer displayed a CE morphology, as judged by their lack of pigmentation and ciliation, and were indistinguishable from photoreceptor cultures *in vitro *[36]. However, photoreceptors do not display their characteristic outer segment morphology *in vitro*, leaving it unclear whether these cells possess the ability to adopt the correct structure *in vivo.* In order to reach a final conclusion on this topic, it would be necessary to perform functional studies to show that CE cells not only are capable of upregulating certain mature retinal markers *in vitro* but also possess the intracellular structures necessary to mature functionally. There are several criteria that are necessary to determine whether a (stem) cell has generated a functional neuron such as a photoreceptor [31, 39]; the cell should be (1) postmitotic, (2) polarized with developed cellular processes, (3) capable of proper electrophysiological activity, and (4) able to communicate with other neurons through synapses. Inoue et al. have shown that adult human CE cells may develop some functional properties of photoreceptor cells; however, their approach required transduction of several key regulator genes of photoreceptor formation [40]. In addition, Jasty et al. recently showed development of functional ionotropic glutamate receptors upon differentiation of adult human CE spheres [41]. There is also evidence that many key properties of retinal cell polarization and function are environment dependent. For instance, in the primate retina, progressive degeneration of the outer segments is observed during the first two weeks after a retinal detachment, and production of outer segments is only initiated after repositioning of the sensory retina in contact with the RPE. Thus, lack of fully functional differentiation of retinal stem/progenitor cells *in vitro* does not predict how these cells may differentiate in a proper *in vivo* environment.

## 6. Stem Cells Should Be Able to Respond to Injury

One final way of assessing the stem cell-potential of CE cells is to examine their response to retinal injury. We hypothesized that if RSCs indeed reside within the CE, they would be activated in eyes suffering from proliferative vitreoretinopathy (PVR) and respond by migrating towards the damaged areas (Table 1) [33]. Retinal injury did induce cell proliferation in the CE, but NSC-markers such as Sox2, Pax6, and Nestin could not be detected in the major parts of the CE, both in injured and uninjured eyes. The only part of the adult human CE that showed some upregulation of NSC markers upon PVR was the most posterior part close to the retinal edge surrounding peripheral cysts. In contrast, we found cellular hyperplasia and Nestin upregulation in the CE of mouse eyes with PVR, suggesting that there may be important species differences in the neural potential of the CE. This is especially of interest since most of the studies supporting the RSC hypothesis in the adult CE have been performed on rodents. Our results partially concur with a recent *in situ *report of 3 human eyes with PVR. In this study, hyperplasia of the CE, forming “neurosphere-like” structures was found. There were no GFAP^+^ cells, but unlike our study, a few rhodopsin^+^ cells were found in the vicinity of the CE [42]. However, finding rhodopsin^+^ cells in the adult CE does not prove that these cells are in fact photoreceptors, as expression of markers usually found in retinal cell types can also be induced in other cells [43]. Future analysis of the adult human CE in patients with retinal damage, including studies using an endoscopic technique during vitreoretinal surgery, would give important new information regarding this controversy [44].

## 7. Future Directions

In order to reach a final ruling on this topic, more knowledge is needed. Results vary greatly between research groups, which may be partly due to the lack of a standardized method for isolation and culturing of CE cells. Variations in culture supplements, protocols for passaging and differentiating cells, and time points at which cells are studied may all affect the cells' phenotype *in vitro*. However, this highlights the problem of solely relying on morphologic and genetic markers for identifying the presence of stem cells. It is possible that cells upregulate certain genes in response to the culture conditions and that this may be misinterpreted as presence of true stem/progenitor cells. Functional studies are thus needed in order to validate the RSC hypothesis. In light of the available evidence, it seems most likely that the adult human CE does not contain *bona fide* NSCs but rather consists of a population of epithelial cells which display a remarkable plasticity *in vitro* reflecting their neuroepithelial developmental origin. Perhaps our current sum of knowledge thus indicates a shift in focus away from studies of the adult human CE for cell-based therapy to restore vision, as stated by Cicero et al. already in 2009 [26].

## Figures and Tables

**Table 1:** Comparison of gene expression profile of four key neural stem cell (NSC) markers.

Gene	WT CE	CE spheres	PVR spheres	SVZ spheres
GFAP	−	−/+	++	+++
Sox-2	−	+	+	++
Nestin	+	+	+	++
Nanog	+	+	+	+

Semiquantitative comparison of RT-PCR expression in adult human ciliary body epithelium (CE) whole tissue samples (WT CE) [32] and cultivated spheres from the adult human CE [32], proliferative vitreoretinopathy (PVR) samples [33], and subventricular zone (SVZ) biopsies [34]. No detectable expression: (−), very low: (−/+), low: (+), middle: (++), and high: (+++) expression.
